# Singlet Fission among Two Single Molecules

**DOI:** 10.1021/jacs.6c05963

**Published:** 2026-06-15

**Authors:** Sumanta Paul, Oleksandr Yampolskyy, Zehua Wu, Klaus Müllen, Thomas Basché

**Affiliations:** † Department of Chemistry, Johannes Gutenberg-Universität, Mainz 55128, Germany; ‡ Max Planck Institute for Polymer Research, Mainz 55128, Germany

## Abstract

Singlet fission (SF) is a photophysical process where a singlet excitation generates two triplet excited states, enhancing exciton multiplication potentially useful for solar energy conversion. Since SF typically outcompetes radiative decay, single molecule studies of SF have remained elusive. Here, we present single molecule spectroscopy of a terrylenediimide (TDI) dimer at room and cryogenic temperatures. By analyzing the stream of photons emitted by single dimers, the rates of formation and decay of SF-born triplet states were determined. We report considerable static and dynamic heterogeneities of the SF process, which are reflected in broad rate distributions as well as the occasional occurrence of delayed fluorescence and rate fluctuations during spin evolution. Cryogenic experiments point to the formation of a coherent multiexciton superposition state that decays into the singlet exciton from which a correlated triplet pair evolves. Our results establish single molecule spectroscopy as a new avenue into mechanistic details of the SF process which often are drowned by ensemble averaging.

## Graphical abstract

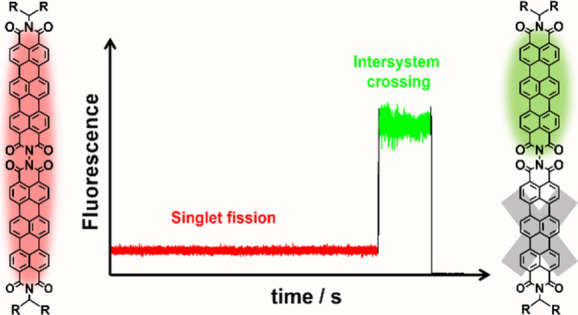

## 1 Introduction

In singlet fission (SF) the absorption of a single photon in molecular assemblies results in the conversion of a singlet exciton into two lower-energy triplet excitons, each localized on adjacent chromophores.^1,2^ SF has garnered significant attention for its potential to enhance the theoretical Shockley–Queisser efficiency limit of single-junction solar cells from 33 to 45%, as well as its emerging applications in quantum information science.^2–6^ SF requires specific conditions: the energy of the lowest singlet excited state (E­(S_1_)) should be roughly twice that of the lowest triplet excited state (E­(S_1_) ≥ 2E­(T_1_)), and the energy of the second triplet state (E­(T_2_)) should exceed 2E­(T_1_).^1,2,7–9^ Spin conservation entails that the initial product of SF is a correlated triplet pair with singlet spin multiplicity, ^1^(T_1_T_1_).^1,2,8,9^ The formation of ^1^(T_1_T_1_) can proceed through either a coherent or an incoherent SF mechanism. In the coherent pathway, a superposition of the delocalized singlet state ^1^(S_1_S_0_) and ^1^(T_1_T_1_) forms immediately after excitation and subsequently dephases to yield ^1^(T_1_T_1_).^1,2,10–15^ In the incoherent mechanism, ^1^(T_1_T_1_) may evolve directly from ^1^(S_1_S_0_) or via a charge-transfer (CT) state that mediates the transition.^1,2,11,16,17^ Following this first step, the interconversion of ^1^(T_1_T_1_) with other triplet-pair states of different spin multiplicities seems to be characteristic for many SF systems. Hereby, a complex ensemble of multiexcitonic triplet-pair states may be involved, distinguished by their degree of exchange coupling, which depends on the orbital overlap and interchromophore distance.^18–24^

Considering molecular dimers, the SF process has been modeled by the following simplified scheme:^9,11,19–23,25,26^

[(S1S0)1⇌(T1T1)1]⇌(T1T1)m⇌(T1+T1) 1

While the ^1^(T_1_T_1_) state may emerge at an ultrafast time scale from ^1^(S_1_S_0_), an equilibrium between the two states or a coherent superposition state may be established as indicated by the square brackets in eq 1. Since spatial dissociation of the triplets is precluded, spin evolution can lead to a spin-entangled mixed state ^m^(T_1_T_1_) of singlet and quintet character as has been reported for several molecular species.^19–22,27^ Eventually, ^m^(T_1_T_1_) may decay into two noninteracting triplet states (T_1_ + T_1_).

Recently, rylene diimides—in particular covalently linked dimers of terrylene diimide (TDI)—have emerged as promising model systems for investigating the SF mechanism.^28–34^ Such dimers enable modulation of electronic coupling as well as exchange coupling through rational linker design. Notably, SF in TDI dimers with covalently linked and slip-stacked geometries was found to initially involve a mixed coherent superposition state with contributions from the ^1^(S_1_S_0_), ^1^(T_1_T_1_) and a charge transfer state.^32–34^ Similar ultrafast state mixing was also reported for a collinear TDI dimer, where two TDI molecules are directly connected at their imide nitrogens (Figure 1b).^30,31^ In this system contributions from a CT state can be neglected and the coherent superposition state with mixed ^1^(S_1_S_0_) and ^1^(T_1_T_1_) character evolved within several tens of femtoseconds. It was suggested that the formation of the coherent superposition state is driven by nonadiabatic vibronic coupling involving low-frequency ^1^(S_1_S_0_) modes and high-frequency ^1^(T_1_T_1_) modes.^31^ Subsequently, noninteracting triplets (T_1_ + T_1_) are generated via an intermediate ^m^(T_1_T_1_) state and then decay to the ground state.^30^

**Figure 1:**
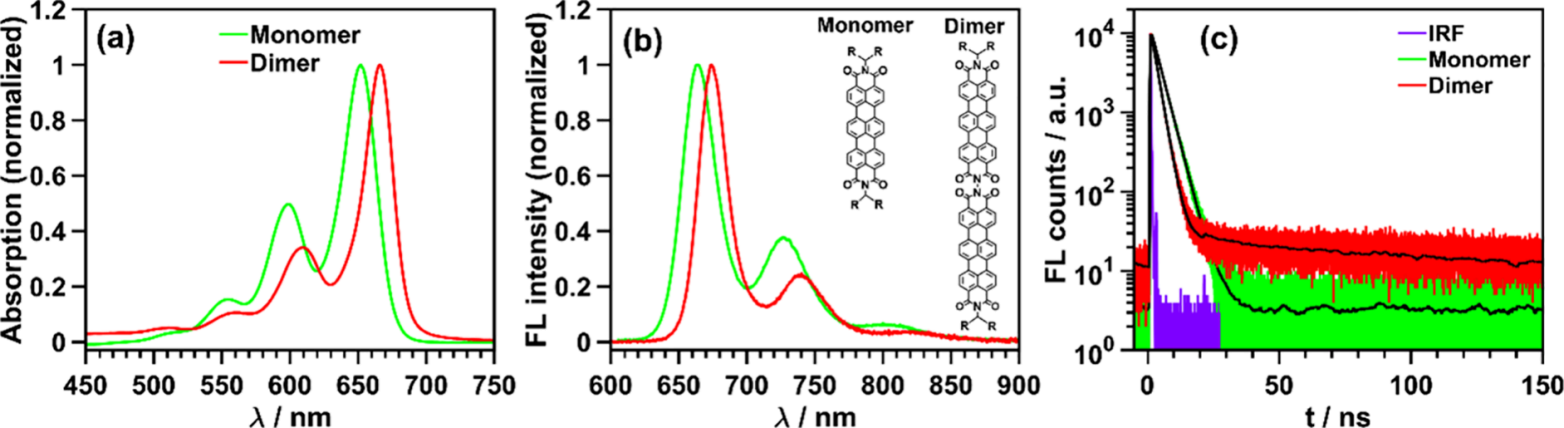
Normalized absorption (a) and fluorescence (b) spectra (λ_exc_ = 594 nm) of the TDI monomer (*c* = 6.4 × 10^–7^ mol/L) and dimer (*c* = 4.7 × 10^–7^ mol/L) in toluene. The molecular structures (*R* = −C_7_H_15_) of the monomer and dimer are shown in the inset of panel (b). Fluorescence decay curves (c) of the TDI monomer and dimer in toluene, recorded after excitation at 600 nm and monitored at their respective emission maxima. The black lines represent the decay models fitted to the data.

Single-molecule spectroscopy studies of SF so far have remained elusive, most probably because SF typically is accompanied by low fluorescence quantum yields. Yet, such studies promise to deliver unique insights into static and dynamic heterogeneities of the SF process which often are drowned in the ensemble average. Moreover, the extremely low concentrations (≈10^–10^ mol/L) used in single-molecule studies largely prevent aggregation of extended π-systems. When looking at a single molecule undergoing transitions between optically bright and dark states, the stream of emitted photons contains information about the temporal succession of these transitions without the need of an external trigger. A powerful way to analyze the underlying kinetics is to measure the fluorescence intensity autocorrelation function which shows photon antibunching at short times and photon bunching due to bright-dark transitions.^35–39^

Here, we employed bulk and single molecule spectroscopy to investigate intramolecular SF in the collinear TDI dimer (Figure 1b). The favorable photophysical properties of TDI for single molecule spectroscopy at room and low temperature^40,41^ and a fluorescence quantum yield in solution of roughly 50% highly qualified this dimer for the intended study. The properties of the emissive state are in accordance with a delocalized S_1_ exciton with a fluorescence lifetime containing contributions from superradiance^42,43^ and SF. By analyzing the fluorescence intensity autocorrelation function of single dimers, we find an overall SF rate which is almost 3 orders of magnitude larger than the intersystem crossing (ISC) rate in TDI monomers while the triplet decay rate almost remains unchanged. Together with the occasional observation of delayed fluorescence, the wide distributions of rate parameters reveal significant differences between individual dimers (static heterogeneity). Moreover, correlated fluctuations of the SF rate and the rate of delayed fluorescence point to slow spin evolution as a signature of dynamic heterogeneity. Strongly broadened excitation spectra of single dimers at 1.4 K support the initial formation of a mixed ^1^(S_1_S_0_) and ^1^(T_1_T_1_) superposition state which decays on an ultrafast time scale. Subsequent creation of the delocalized pure ^1^(S_1_S_0_) state rationalizes slow overall SF as well as the large fluorescence quantum yield.

## 2 Experimental Section

### 2.1 Synthesis

The synthesis of the TDI monomer^44^ and the TDI dimer^30^ followed procedures previously described in the literature (Figures S1–S4).

### 2.2 Bulk Absorption and Fluorescence Spectroscopy

Absorption and emission spectra were recorded using a Duetta absorbance and fluorescence spectrometer (Jobin-Yvon) with a sensitivity extending up to 1100 nm. Fluorescence decays were acquired by time-correlated single photon counting (TCSPC) measurements on a modified Fluorolog-3 spectrofluorometer (Jobin-Yvon). The setup was equipped with a pulsed fiber laser (YSL SC-OEM + YSL VLF) and a hybrid detector (PicoQuant, PMA Hybrid 50), both connected to a TCSPC module (PicoQuant, PicoHarp 300) yielding a time resolution of ∼300 ps.

### 2.3 Single Molecule Spectroscopy

Thin-film samples for single molecule measurements were prepared by spin-coating toluene/zeonex 330R solutions containing TDI/TDI dimer at concentrations ranging from 10^–10^ to 10^–11^ M on top of cleaned glass cover slides. Zeonex, a nonpolar hydrocarbon polymer, was chosen because of its low dielectric constant (*e* = 2.5) which is close to that of toluene and should prevent contributions of charge transfer states to SF as was reported in the literature.^29^ Single molecule measurements at room temperature were conducted with a home-built confocal fluorescence microscope. Excitation was provided either by a fiber-coupled continuous wave (cw) solid-state laser (Coherent OBIS, 594 nm) or, in the case of pulsed operation, by a pulsed white-light fiber laser (SC-OEM, YSL Photonics) with a variable optical filter (YSL VLF) tuned to ∼594 nm. The laser beam was collimated and focused onto the sample plane using an oil-immersion objective (Zeiss, Plan-Apochromat 63×, NA = 1.4) after passing through a 594 nm band-pass filter and reflecting off an 80T:20R beam splitter. The laser power at the sample was ∼5 μW, corresponding to an excitation intensity of ∼1 kW/cm^2^, with argon gas flowing continuously to minimize photobleaching of TDI monomers and dimers. Fluorescence was collected through the same objective, with the excitation light blocked by a 615 nm long-pass filter in the detection path. The fluorescence light was then split into two paths by a 50T:50R beam splitter. The light in one path was further divided and sent to two avalanche photodiodes (APDs) in a Hanbury-Brown Twiss configuration to detect photon arrival times, which were recorded using a HydraHarp 400 TCSPC module (PicoQuant). The light in the second path was directed to a spectrograph (Acton Spectra Pro 300i, resolution ∼25 cm^–1^) equipped with an EM-CCD camera (ProEM HS, Teledyne Princeton Instruments).

Measurements at 1.4 K were performed using a home-built variable-temperature confocal microscope. To record fluorescence emission spectra, excitation was provided by the same cw or pulsed lasers used in the room-temperature experiments. The excitation light was focused onto the sample with a microscope objective (Melles Griot, 60×, NA 0.85) mounted inside an optical cryostat and immersed in liquid helium. Fluorescence emission was collected by the same objective, filtered with a 615 nm long-pass filter, and split by a 50T:50R beamsplitter. One fraction was directed to two APDs in a Hanbury–Brown–Twiss configuration as described above. The other fraction was dispersed by a spectrograph (Acton Spectra Pro 500i, resolution ∼20 cm^–1^) and detected with an EM-CCD camera (Newton, Andor). Fluorescence excitation spectra were acquired using a tunable ring dye laser (Coherent 899-01) operated in broadband mode (3 GHz bandwidth) with a DCM dye solution providing a scan range of 620–670 nm. Red-shifted fluorescence was collected using a 685 nm long-pass filter and detected by the two APDs. Excitation intensities during the scans ranged from 3 to 152 W/cm^2^.

### 2.4 Fluorescence Intensity Autocorrelation Analysis and Estimation of the Rates k_ISC_, k_SF_, and k_T_

Fluorescence intensity autocorrelation functions were generated from the photon arrival times recorded with the HydraHarp 400. Fitting of the correlation functions was performed using home-written MATLAB routines. For the theoretical treatment of the model, including the derivation of the rate constants, we refer to the literature.^45^ In our analysis, the triplet population rate is denoted as k_ISC_ for the TDI monomer and k_SF_ for the dimer, reflecting their different physical origins (intersystem crossing vs singlet fission). Mathematically, both processes are represented by the same parameter in the fitting model.

The fitting parameters λ_1_, λ_2_, and C obtained from eq 6 are given by

$$\lambda_{1}=\frac{1}{2}\left(a+d-\sqrt{{\left(a-d\right)}^{2}+4\mathrm{bc}}\right)$$ 2

$$\lambda_{2}=\frac{1}{2}\left(a+d+\sqrt{{\left(a-d\right)}^{2}+4\mathrm{bc}}\right)$$ 3

$$C=-\frac{\lambda_{1}\left(1+\lambda_{2}/f\right)}{\left(\lambda_{1}-\lambda_{2}\right)}$$ 4

*a*, *b*, *c*, *d* and *f* are given by the following expressions:

$$a=-(k_{\mathrm{exc}}+k_{T}),\;b=\left(\frac{1}{\tau_{\mathrm{FL}}}-k_{\mathrm{ISC}/\mathrm{SF}}-k_{T}\right),\;c=k_{\mathrm{exc}}$$

$$d=-\frac{1}{\tau_{\mathrm{FL}}},\mathrm{and}\;f=k_{T}$$

## 3 Results

### 3.1 Bulk Absorption and Fluorescence Measurements

The steady-state absorption and emission spectra of the TDI monomer and dimer in toluene are presented in Figure 1a,b. The absorption spectrum of the TDI monomer features three well-resolved bands at 652, 599, and 552 nm, corresponding to the 0–0, 0–1, and 0–2 vibronic transitions, respectively. In case of the TDI dimer these bands are red-shifted by about 12–13 nm. The fluorescence spectra of both species closely mirror their absorption spectra, displaying the same vibronic band signatures which are red-shifted by 11–12 nm for the dimer (Table 1). Notably, the relative intensities of the 0–0 and 0–1 bands differ significantly between the dimer and monomer in both absorption and emission spectra (Figure 1a,b). In particular, the TDI dimer shows an increased ratio of the intensity of the 0–0 bands (*I*_0–0_) to the 0–1 (*I*_0–1_) bands compared to the monomer. The increase of the *I*_0‑0_/*I*_0‑1_ ratio in the dimer reports about exciton delocalization and signals the formation of a coherent state through electronic coupling.^42,43^ In accordance with the SF literature, we have denoted this state with ^1^(S_1_S_0_) keeping in mind that we are dealing with a delocalized S_1_ exciton state. Because the HOMO and LUMO possess orbital nodes at the imide linkage, through bond conjugation can be largely ruled out. In addition, orbital overlap is negligible due to the almost orthogonal orientation of the TDI units. Assuming through space electrostatic dipole–dipole coupling (see Supporting Information for details), the coupling strength V was estimated to be 240 cm^–1^, which predicts a red-shifted dimer emission maximum at 674 nm, in good agreement with the experimental observation of 675 nm.

**Table 1:** Photophysical Parameters of the TDI Monomer and Dimer in Bulk Toluene Solution

system	λ_ab_^max^/nm	λ_em_^max^/nm	τ_FL_/ns	τ_del_/ns	ϕ	k_FL_/10^8^s^–1^	*k*_rad_/10^8^s^–1^	k_SF_/10^8^s^–1^
monomer	652 ± 1	663 ± 1	3.4 ± 0.1		0.7 ± 0.06	2.9 ± 0.1	2.1 ± 0.2	
dimer	665 ± 2	675 ± 2	2.2 ± 0.04	80 ± 17	0.64 ± 0.05	4.5 ± 0.08	2.9 ± 0.2	0.8

Fluorescence decay curves of the TDI monomer and dimer in toluene are shown in Figure 1c. The TDI monomer exhibits a single exponential fluorescence decay with a lifetime of 3.4 ns (Table 1). In contrast, the TDI dimer displays a biexponential fluorescence decay, featuring a dominant short lifetime component (τ_FL_) of 2.2 ns (∼95%) and a minor long lifetime component (τ_del_) of 80 ± 17 ns (∼5%). The long lifetime component which was also found at the single molecule level (see below) is attributed to delayed fluorescence which was not reported in a previous investigation.^30^ The substantial shortening of the short lifetime component from 3.4 ns in the monomer to 2.2 ns in the dimer is accompanied by only a slight decrease of the fluorescence quantum yield from 0.7 (monomer) to 0.64 (dimer). These numbers are likely influenced by the opposing effects of SF on the one side and electronic coupling on the other side. The nonradiative process of SF is expected to lead to a decrease of the fluorescence lifetime and quantum yield. While electronic coupling also leads to a decrease of the lifetime, this is due to an increase of the radiative rate (superradiance) which should be accompanied by an increase of the quantum yield.^43^ The radiative rate is given by *k*_rad_ = Φ_FL_ × k_FL_, with k_FL_ = (τ_FL_)^−1^. For the TDI monomer we obtain *k*_rad_ = 2.1 × 10^8^ s^–1^. For the TDI dimer we find *k*_rad_ = 2.9 × 10^8^ s^–1^, in good agreement with an independent estimate based on the coherence enhancement number N_coh_ (see Supporting Information for details). The fluorescence decay rate is given by all processes which lead to the decay of the S_1_ state. In case of the TDI monomer this reads: k_FL_ = *k*_rad_ + k_IC_ + k_ISC_, with k_IC_ and k_ISC_ being the S_1_–S_0_ internal conversion rate and the intersystem crossing rate, respectively. Since k_ISC_ is much smaller than all other rates (see below), it can be safely neglected here. For the TDI monomer we find k_IC_ = 8 × 10^7^ s^–1^. Assuming that the IC rate does not change in the dimer, the radiative rate and IC rate add up to a value of 3.7 × 10^8^ s^–1^ which is smaller than the total fluorescence decay rate of the dimer, k_FL_ = 4.5 × 10^8^ s^–1^. The difference (8 × 10^7^ s^–1^) is assigned to the additional contribution from SF as will be corroborated and more rigorously quantified by the single molecule measurements.

### 3.2 Single Molecule Measurements at Room Temperature

Figure 2a,c present the fluorescence intensity time-trace and spectrum of a representative TDI monomer molecule, respectively. The fluorescence spectra of single TDI molecules in Zeonex are nearly identical to those observed in bulk toluene solution, with a distribution of peak positions (Figure S5a and Table 2). The 13 nm spectral blue shift in the Zeonex film compared to toluene is attributed to the different environments.

**Figure 2:**
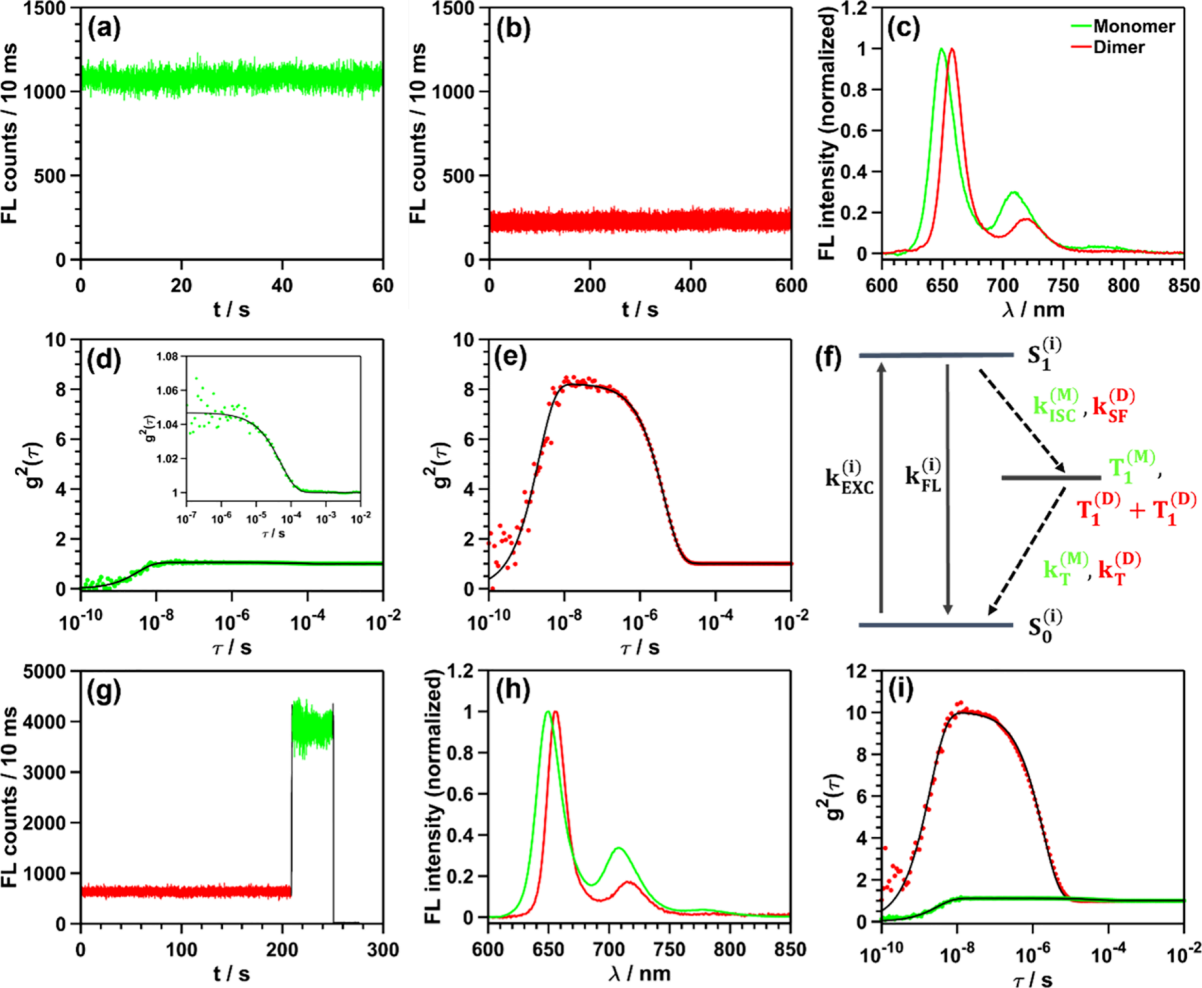
Fluorescence intensity time-traces of a single TDI monomer (a) and a single TDI dimer (b) and the corresponding fluorescence spectra (c). In accordance with the bulk solution data (Figure 1b), the fluorescence spectrum of the dimer is red-shifted with respect to the monomer and the intensity of the vibronic sideband is decreased. Fluorescence intensity autocorrelation functions *g* ^2^(τ) of the TDI monomer (d) and dimer (e). In the inset of panel (d) the bunching part of the correlation function is displayed on an expanded scale. In panel (f) a scheme for the photophysical transitions is presented to stress the analogy of transitions into a dark state in both the monomer (M) and dimer (D). Here k_exc_, k_FL_, k_ISC_, k_SF_ and k_T_ represent excitation, fluorescence, intersystem crossing, singlet fission and triplet decay rates. Fluorescence time trace (g) of another single TDI dimer. After ∼210 s a strong increase in the count rate is observed due to photobleaching of one of the monomers. The fluorescence spectra (h) and correlation functions (i) during the red and green phases. All data shown in this figure have been recorded using continuous-wave (cw) laser excitation.

**Table 2:** Photophysical Parameters Derived from Single Molecule Measurements

system	λ_FL_^max, avg^/nm	τ_FL_^avg^/ns	τ_del_^avg^/ns	k_FL_^avg^/10^8^s^–1^	k_ISC_^avg^/10^5^s^–1^	k_SF_^avg^/10^8^s^–1^	k_T_^avg^/10^4^s^–1^
monomer^a^	650 ± 4	3.6 ± 0.3		2.8 ± 0.2	2.5 ± 1.3		1.8 ± 0.4
dimer^a^	659 ± 4	2.3 ± 0.3		4.3 ± 0.6		1.3 ± 1	2.6 ± 1
dimer^b^	661 ± 5	2.2 ± 0.4	24.3 ± 13	4.6 ± 0.8		0.6 ± 0.3	2.2 ± 0.4
monomer^c^	654 ± 4	3.3 ± 0.6		3.0 ± 0.6	7.0		1.7 ± 0.7

a Data derived from experiments under both cw and pulsed excitation.

b Dimers with prompt and delayed fluorescence.

c Monomers formed after bleaching of one TDI unit in the dimers.

Since the internal photophysical transitions control the sequence of photons emitted, the fluorescence intensity autocorrelation function *g* ^2^(τ) provides an ideal tool for measuring the rates of these transitions for immobilized single molecules.^35,38–40^ In terms of photon counts or intensities, *g* ^2^(τ) is defined as^45,46^

$$g^{2}(\tau )=\frac{\left\langle I(t)I(t+\tau )\right\rangle }{\langle I(t)\rangle^{2}}$$ 5

Exemplarily, *g* ^2^(τ) of a TDI monomer is shown in Figure 2d exhibiting the typical features resulting from the photon statistics of a single organic dye molecule. To extract the transition rates for the TDI monomer from *g* ^2^(τ), we model it as an effective three-level system (Figure 2f), following established methods in the literature.^35,37,38,45^ In our experiments, the molecules are excited nonresonantly into a higher energy vibronic level of the S_1_ state at room temperature. Under these conditions coherences can be ignored, simplifying the analysis to a system of rate equations that describe the transitions between the energy levels. Using this three-state model, *g* ^2^(τ) is given by the following expression:^45^

$$g^{2}(\tau )=-(1+C)e^{\lambda_{1}\tau }+{\mathrm{Ce}}^{\lambda_{2}\tau }+1$$ 6

Here, C, λ_1_, and λ_2_ represent the contrast, rise (antibunching component), and decay (bunching component) parameters of the correlation function, respectively. Since the typical excitation rates (*k*_exc_ = 4 × 10^5^ s^–1^) were significantly lower than the fluorescence decay rate (k_FL_∼10^8^ s^–1^), λ_1_ ≈ k_FL_. The above parameters were then used as inputs to simultaneously calculate the ISC rate constant (k_ISC_), the triplet decay rate (k_T_) and the fluorescence lifetime (τ_FL_) in the TDI monomer through global analysis (see Experimental Section for details).

The data of the representative TDI monomer (Figure 2d) gave rise to an ISC rate of k_ISC_ = 3.3 × 10^5^ s^–1^, a triplet decay rate of k_T_ = 1.9 × 10^4^ s^–1^ and a fluorescence lifetime of τ_FL_ = 3.2 ns. The distributions and average values of these parameters for TDI monomers are given in Figure S6 and Table 2. The fluorescence lifetime distribution peaks at 3.6 ns which is close to the value observed for TDI monomers in toluene solution (Table 1). Having the fluorescence decay rate and the triplet kinetics of the TDI monomer at our disposal, we have an ideal reference system to compare to the behavior of the TDI dimer.

In Figure 2b,c the fluorescence time trace and spectrum of a single TDI dimer are shown. In accordance with the bulk solution data (Figure 1b), the fluorescence spectrum of the dimer (Figures 2c and S5b) is red-shifted with respect to the monomer and the intensity of the vibronic sideband is decreased, corroborating the presence of the delocalized S_1_ state in the dimer and directly signaling electronic coupling without reference to any photophysical rate parameters.

The autocorrelation function of the dimer (Figure 2e) reveals a much higher contrast than that of the TDI monomer and the bunching part is shifted to shorter times. The correlation function could also be well fitted with eq 6 (Figure 2e). Accordingly, we will provisionally approximate the dimer as an effective 3-level system undergoing transitions between optically bright and dark states. In line with the 3-level description of the monomer, we have defined the transition rates of the dimer as depicted in the scheme in Figure 2f. k_SF_ denotes the overall formation rate of the SF-born triplets. This process may involve several intermediate states to be discussed later. The decoupled triplets then decay with the rate k_T_. Solving again for the 3-level system population dynamics, we find for the dimer in Figure 2e, k_SF_ = 2.9 × 10^8^ s^–1^, k_T_ = 2.7 × 10^4^ s^–1^ and a fluorescence lifetime of τ_FL_ = 2.1 ns.

The characteristic differences between TDI dimers and monomers are nicely reproduced in the sequence of events once one of the monomers bleaches in a TDI dimer (Figure 2g). In the fluorescence time trace (Figure 2g), the initial fluorescence count rate is low (red phase) and switches suddenly to a much higher level at ∼210 s (green phase). The count rate has increased appreciably and remains stable until the signal irreversibly drops to the background level. The fluorescence spectrum (Figure 2h) during the green phase is blue-shifted (compared to the red phase) and shows an increased intensity of the vibronic sideband (0–1 transition). These observations indicate that during the red phase an intact TDI dimer is active, while the data in the green phase originate from a TDI monomer. Accordingly, at the end of the red phase one of the TDI monomers has selectively photobleached which is a quite common event when studying individual multichromophoric compounds.^47^ The correlation functions in the two phases (Figure 2i) show the characteristic differences in correlation contrast to be expected for the dimer (red) and monomer (green), respectively. In the red phase, k_SF_ = 3.5 × 10^8^ s^–1^ is found being significantly higher than the ISC rate k_ISC_ = 8.9 × 10^5^ s^–1^ obtained for the monomer in the green phase. The k_T_ values for the red and green phase are 5.2 × 10^4^ s^–1^ and 1.6 × 10^4^ s^–1^, respectively. The fluorescence lifetimes in the two phases are 1.9 ns (red) and 2.9 ns (green), again substantiating the assignment to dimer and monomer emission. This behavior was consistently observed for TDI dimers where photobleaching occurred (Table 2).

As is evident from the fluorescence intensity time-traces (Figure 2a,b,g), TDI dimers exhibit significantly lower count rates than monomers. Since the fluorescence lifetimes remain unchanged from toluene solution to Zeonex for both monomers and dimers, the quantum yields at the bulk and single molecule level are expected to be similar in both matrices. Accordingly, the smaller count rates in the dimers do not reflect a decrease of the fluorescence quantum yield but are primarily due to early saturation of the emission rate because of efficient population of the triplet bottleneck.

The distributions of k_SF_ and k_ISC_ for TDI dimers and monomers, respectively, are presented in Figure 3a. The average value of k_SF_^avg^ = 1.3 × 10^8^ s^–1^ (Table 2) is more than 500 times larger than the average ISC rate in the monomer indicating efficient population of triplet states. When comparing k_T_ in the dimer and monomer (Figure 3b), it is seen that the bulk of the dimer distribution largely overlaps the monomer distribution with k_T_^avg^ = 2.6 × 10^4^ s^–1^. The dimers with larger k_T_ values may represent cases where the decay is accelerated by triplet–triplet annihilation assuming that preferentially two triplet states (T_1_ + T_1_) are generated.^21^

**Figure 3:**
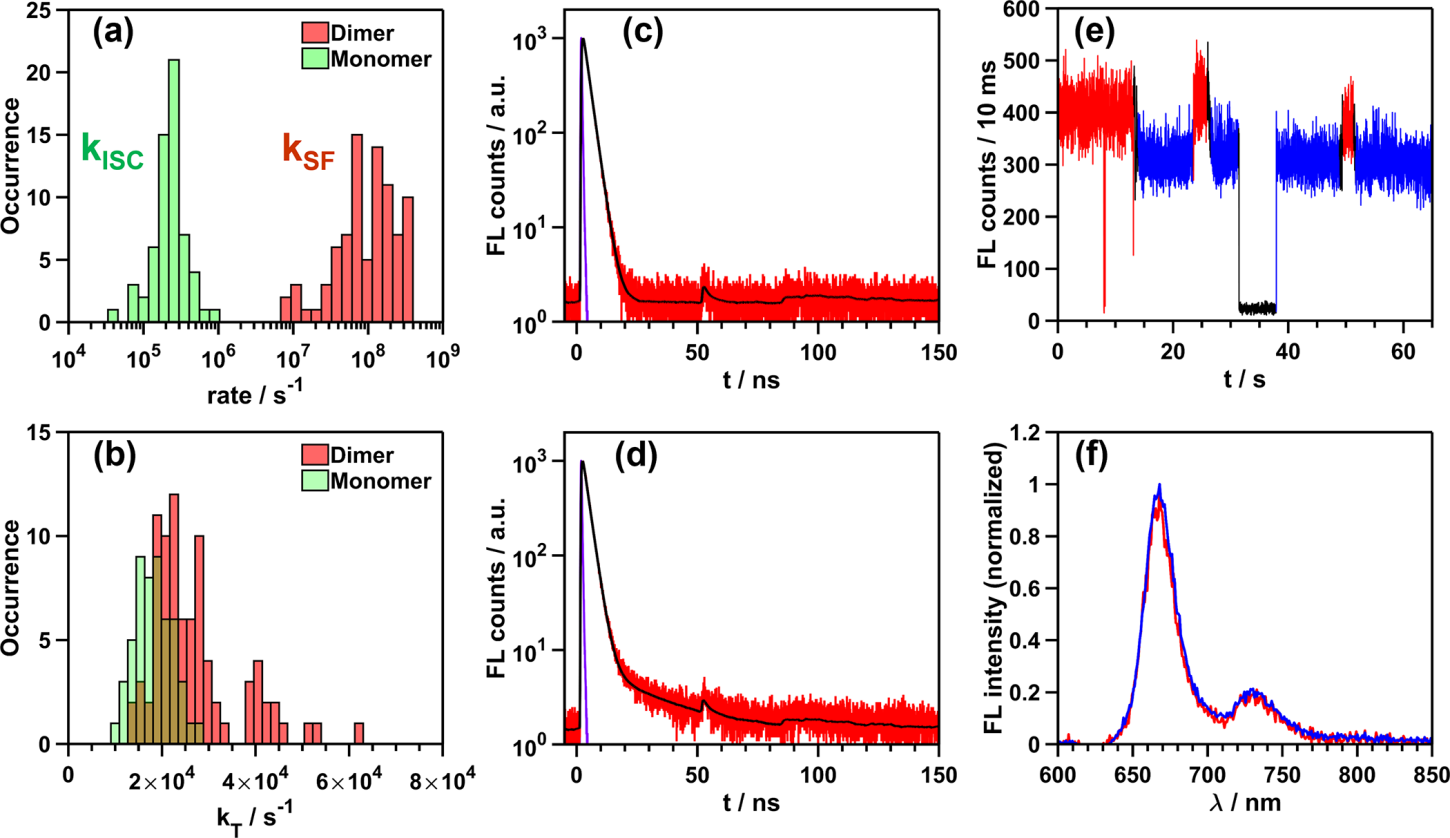
(a) Distributions of k_SF_ and k_ISC_ for TDI dimers (red) and monomers (light green). (b) Distributions of k_T_ for TDI dimers (red) and monomers (light green). The average values are given in Table 2. The distributions are derived from data recorded under both cw and pulsed excitation. Fluorescence decay curves of two single TDI dimers in zeonex (c, d). The dimer in (c) gave rise to a single exponential fluorescence decay (prompt fluorescence) with a lifetime of 2.4 ns. In contrast, the dimer in (d) showed a biexponential decay with contributions from prompt fluorescence (τ_FL_ = 2.4 ns, 94%) and delayed fluorescence (τ_del_ = 24.5 ns, 6%). Fluorescence time trace (e) of a single TDI dimer exhibiting slow fluctuations of the count rate between two levels (red and blue). Fluorescence spectra (f) recorded during the red and blue phases. No differences in peak position and spectral shape were observed, demonstrating that the electronic coupling remained unchanged during the entire sequence.

So far, the discussion has referred to data obtained with continuous-wave (cw) excitation. In addition, experiments with pulsed excitation were performed. In Figure 3c,d, the fluorescence decay curves of two individual TDI dimers are displayed. In one case (Figure 3c) a single exponential decay was observed with a fluorescence lifetime of 2.4 ns, while in the other case (Figure 3d) the decay was biexponential which we assign to prompt (τ_FL_ = 2.4 ns (94%)) and delayed fluorescence (τ_del_ = 24.5 ns (6%)). Such biexponential decays were observed for 20% of the dimers. The single molecule delayed fluorescence lifetimes appeared to be shorter than the bulk value in toluene and were distributed between 10 and 40 ns (see Figure S7). We note, however, that due to signal-to-noise limitations in the single molecule measurements, decays with longer lifetimes may have escaped detection. The data indicate that the occurrence of delayed fluorescence and its contribution to the total decay strongly varies from dimer to dimer. Considering, that delayed fluorescence is a thermally activated process, a potential barrier has to be overcome to induce triplet fusion. The barrier height is thought to be determined by the conformation of a given dimer and the interaction with its particular local environment. Since conformation and local environment are widely distributed in a disordered polymer host, there will also be a wide distribution of barrier heights. In the Zeonex host this leads to a fraction of 20% of dimers which showed delayed fluorescence. By lowering the temperature (see below), delayed fluorescence completely vanishes.

In Figure S8 the distributions of prompt fluorescence lifetimes of single TDI dimers obtained from the correlation analysis after cw excitation and from the fluorescence decay curves after pulsed excitation are presented. The average values (2.2 and 2.3 ns, respectively) agree within experimental error and match the bulk value (2.2 ns), confirming the reliability of the photophysical parameters derived from the multiparameter correlation analysis.

The distributions presented in Figure 3a,b as well as the observation of varying fluorescence decay behavior have shown that the photophysics varies appreciably from dimer to dimer. In addition to these static heterogeneities, we have observed dynamic heterogeneities referring to changes in the behavior of a given dimer in the course of time. In the example shown in Figure 3e, the fluorescence count rate is fluctuating on a slow time scale between two levels. The reversible transition to a dark state at ∼30 s will not be considered further. The fluorescence spectra do not show any differences in position and shape between the red and blue phases (Figure 3f) clearly demonstrating that the electronic coupling does not change throughout the whole sequence. During the red as well as the blue phases the fluorescence decays are biexponential revealing prompt and delayed fluorescence. The prompt fluorescence lifetime – dominated by the radiative rate – remains constant during the whole time-trace (τ_FL_ = 2.2 ± 0.04 ns). In contrast, the lifetime of the delayed fluorescence is different in the red and blue phases. In the red phase the average value is τ_del_ = 12 ± 1.2 ns while in the blue phase we find τ_del_ = 17 ± 2.8 ns. For later comparison, we convert the delayed fluorescence lifetimes into rates (red phase: k_del_ = 8.2 ± 0.8 × 10^7^ s^–1^; blue phase: k_del_ = 5.9 ± 1.0 × 10^7^ s^–1^). In addition, k_SF_ has been determined from the correlation function for both phases (red phase: k_SF_ = 4.2 ± 0.4 × 10^7^ s^–1^; blue phase: k_SF_ = 6.8 ± 0.7 × 10^7^ s^–1^). When comparing k_del_ in the red and blue phase, it is seen that it is smaller in the blue phase. In contrast, k_SF_ is larger in the blue and smaller in the red phase. Within the error margins the sum of both rates (k_del_ + k_SF_) in each of the two phases is constant indicating that the two rates are not changing independently. Moreover, with an increased (nonradiative) rate k_SF_ in the blue phases, the fluorescence count rate roughly drops by the same factor (Figure 3e).

In Figure S9, distributions and average values of k_SF_ and k_T_ are presented for dimers which exhibited either only prompt fluorescence or both prompt and delayed fluorescence after pulsed excitation. When delayed fluorescence was observed, the average k_SF_ value was smaller than for dimers which showed only prompt fluorescence. In contrast, the average k_T_ value did not depend on the absence or presence of delayed fluorescence. Interestingly, the photostability of dimers which showed delayed fluorescence was larger than for those with prompt fluorescence only. Apparently, a more efficient back transfer into the ^1^(S_1_S_0_)-state protects the molecules from photobleaching which is thought to occur more efficiently from the triplet state.^48^

### 3.3 Measurements at Cryogenic Temperature (*T* = 1.4 K)

To address the notion of a coherent superposition state, we also performed measurements at 1.4 K at which the effects of thermal broadening and activation are largely suppressed. The fluorescence lifetime at 1.4 K (Figure 4a) was basically the same as at room temperature. In no case, however, delayed fluorescence was observed. Yet, in the low temperature measurements a reliable analysis of the intensity correlation function was prevented, because the excitation rate could not be determined accurately. Since the fluorescence lifetime did not change, it is reasonable to assume that the singlet fission rate (k_SF_) also remained unaffected.

**Figure 4:**
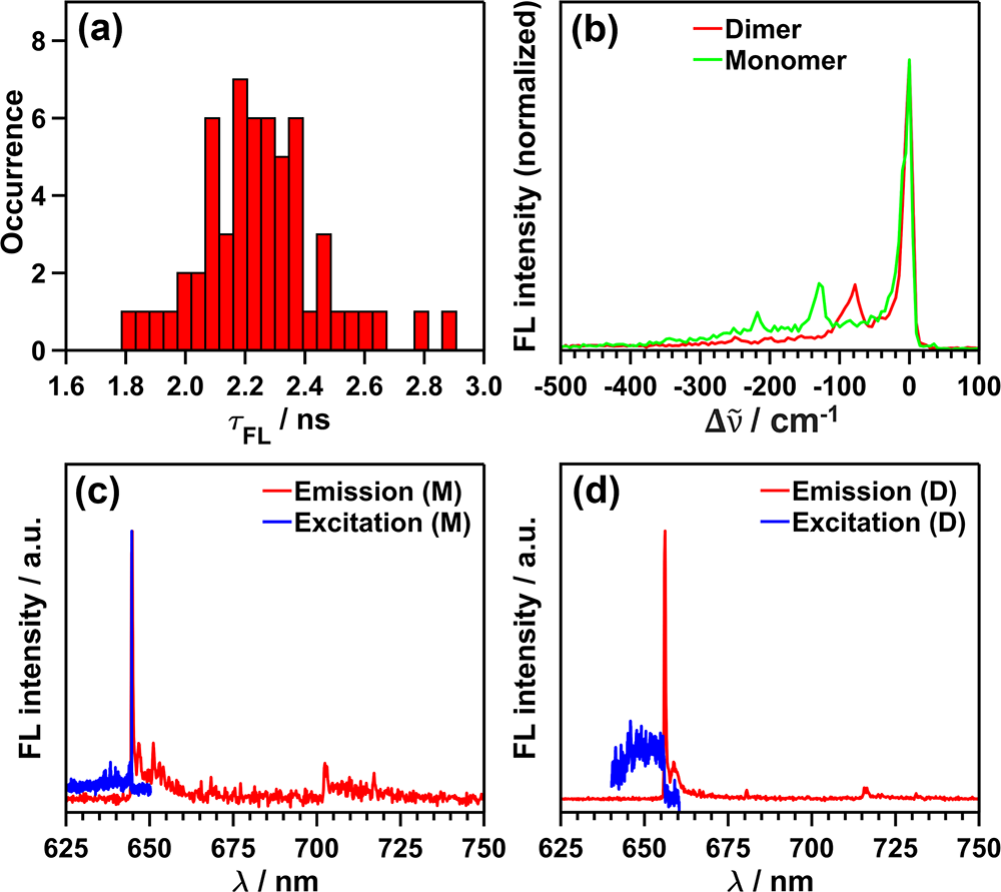
(a) Fluorescence lifetime (τ_FL_) distribution of the TDI dimer derived from fluorescence decay measurements at 1.4 K. The average lifetime calculated from the distribution (τ_FL_^avg^ = 2.3 ± 0.2 ns) is identical to the bulk and single molecule values obtained at room temperature. (b) Representative fluorescence spectra of a single TDI monomer (green) and dimer (red). Fluorescence emission (red) and excitation (blue) spectra of a TDI monomer (c) and a dimer (d).

Furthermore, we have measured fluorescence emission and excitation spectra at 1.4 K of monomers and dimers. As seen in Figure S10b, the monomers exhibit a sharp and intense [0,0] zero-phonon line (ZPL) and several well-resolved vibronic transitions. In the dimers a low-frequency mode at 80 cm^–1^ dominates the vibronic structure. A comparison of the monomer and dimer spectra in the low-frequency region (Figure 4b) highlights the emergence of this intermolecular mode, previously observed by femtosecond stimulated Raman spectroscopy (FSRS) spectroscopy and tentatively assigned to a breathing mode with torsional contributions.^31^ In Figure 4c the fluorescence emission and excitation spectra of a TDI monomer are displayed. In both types of spectra, resonant [0,0]-ZPLs are observed. The observed line width of the ZPL in the excitation spectrum is limited by the laser bandwidth (3 GHz), while the true line width should be close to the lifetime limit (50 MHz). In contrast, TDI dimers exhibit a markedly different behavior. While the emission spectrum still showed a sharp ZPL and the characteristic 80 cm^–1^ mode, the excitation spectrum typically consisted of only a broad band (Figure 4d). In rare cases, a very weak ZPL could be discerned at the low energy edge of the broad band. For an analogous *N*–*N*-coupled collinear Perylenediimide-dimer, in which no SF occurs, [0,0]-ZPLs were observed in both absorption and emission.^43^ The lack of symmetry between excitation and emission spectra in the TDI dimer clearly indicates that the absorbing and emitting states are different. The absence of a ZPL in the excitation spectrum is a consequence of strong electron–phonon coupling which appears to be characteristic of the initially photoexcited state. In agreement with previous suggestions,^30,31^ we assume that this state is a coherent (^1^(S_1_S_0_) ↔ ^1^(T_1_T_1_)) superposition. In addition, our data let us to conclude that ^1^(S_1_S_0_) evolves from the decaying superposition state as outlined in the next section.

## 4 Discussion

Considering our single molecule and pertinent literature data, we propose the simplified scheme in Figure 5 for the formation and decay of SF born triplet states. This model assumes the initial formation and decay of a coherent ^1^(S_1_S_0_) ↔ ^1^(T_1_T_1_) superposition state, strongly supported by our low temperature single molecule measurements. As a next step, the superposition state decays either directly into ^1^(T_1_T_1_) or into ^1^(S_1_S_0_). In a recent study a similar scheme has been suggested for SF in a rubrene crystal.^49^ In this case the system was probed by ultrafast transient absorption spectroscopy, and the decay of the coherent superposition state was treated as internal conversion/vibrational relaxation occurring on an 80 fs time scale. The corresponding rates *k*_dec_ (Figure 5) are not accessible in our experiments. The high fluorescence quantum yield of the TDI dimer suggests that the superposition state decays predominantly into ^1^(S_1_S_0_). Regarding the pure ^1^(S_1_S_0_) state, we emphasize that the data for this delocalized singlet state did not show any admixture of the ^1^(T_1_T_1_) state. The pure ^1^(S_1_S_0_) state either decays to S_0_ or ^1^(T_1_T_1_) is formed. Next, the spin pure ^1^(T_1_T_1_) state can develop into an intermediate mixed spin state ^m^(T_1_T_1_) which in turn either leads to delayed fluorescence (see below) or to independent triplets (T_1_ + T_1_) eventually decaying to S_0_. In the single molecule correlation measurements, however, the different dark triplet states depicted in Figure 5 appear as a single dark state and cannot be distinguished. Consequently, the rate constant k_SF_, provisionally defined in Figure 2f, has to be understood as the overall rate-determining SF step, encompassing all processes that convert the fluorescent ^1^(S_1_S_0_) state into uncoupled triplets (T_1_ + T_1_). The average k_SF_ value of 1.3 × 10^8^ s^–1^ for TDI dimers in zeonex is close to the formation rate of uncorrelated triplets (8 × 10^7^ s^–1^) deduced from the modeling of nsTA data for the TDI dimer in chlorobenzene.^30^ Notably, the latter value is basically identical to the value obtained from our bulk solution data (Table 1).

**Figure 5:**
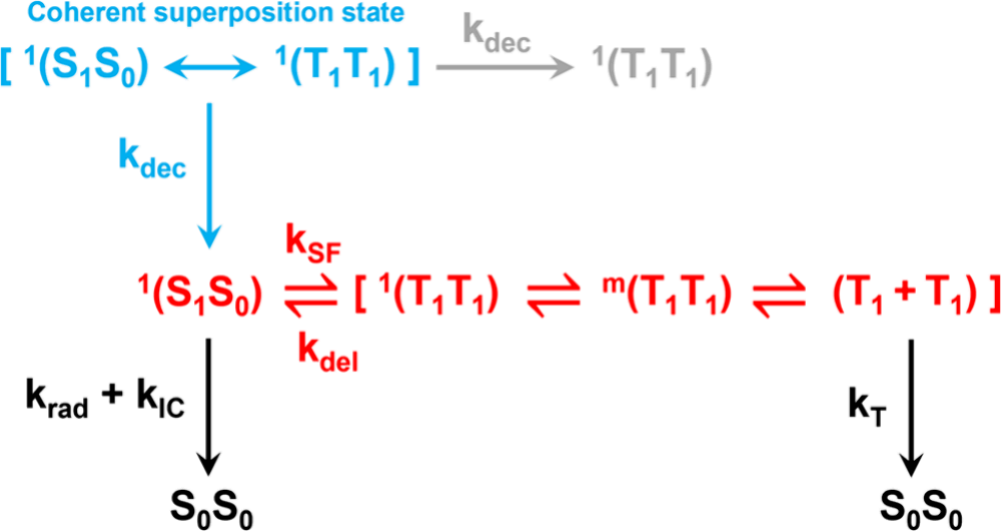
Scheme to describe photophysical transitions and spin evolution in the TDI dimer. The model assumes the initial formation of a coherent superposition state, ^1^(S_1_S_0_) ↔ ^1^(T_1_T_1_). As a next step, the superposition state decays into either ^1^(S_1_S_0_) or directly into ^1^(T_1_T_1_). The corresponding rates *k*_dec_ are not accessible in our experiments. From the pure ^1^(S_1_S_0_) state, either the ^1^(T_1_T_1_) or the singlet ground state (S_0_) are populated. The spin-pure ^1^(T_1_T_1_) state may evolve into an intermediate mixed-spin state ^m^(T_1_T_1_). Since the various dark state transitions in the triplet manifold may have an impact on the rates of SF and delayed fluorescence, these rates may reflect the sum of several processes. The independent triplets (T_1_ + T_1_) eventually decay to S_0_.

Time resolved EPR spectroscopy had disclosed that the spin multiexciton state ^m^(T_1_T_1_) of the TDI dimer is composed of a mixture of singlet and quintet states and that without the presence of a magnetic field the formation of a ^3^(T_1_T_1_) state can be neglected and almost all triplet formation accounted for by triplet dissociation.^30^ Accordingly, out of the mixed ^m^(T_1_T_1_) spin state uncoupled triplets are formed. Since we have found the distribution of triplet decay rates k_T_ to be bimodal (Figure 3b), the fraction with a faster decay than observed for the TDI monomer seem to reflect triplet–triplet annihilation.

In covalently coupled, spatially isolated dimers the triplets cannot diffuse away. Therefore, spin dephasing might be prevented, in which case the triplets preferentially annihilate back to the singlet ground state S_0_.^18,21,22,34^ In our case, however, we clearly have observed a correlation decay on a time scale of the triplet lifetime. Generally, it is thought that structural changes are needed to induce spin dephasing. In the TDI dimer, the monomers are twisted against each other by almost 90° which leads to small orbital overlap and weak exchange coupling. In our low temperature measurements, a prominent low frequency intermolecular mode at 80 cm^–1^ has been observed. We assume that such a mode can modulate the overlap and exchange coupling between the two TDI units.^50^ This appears to be a route to induce spin dephasing and generate uncoupled triplets.

Besides dissociating into uncoupled triplets, the mixed spin state can revert back to the ^1^(S_1_S_0_) state by triplet fusion leading to delayed fluorescence. Delayed fluorescence was observed for approximately 20% of the dimers at room temperature but disappeared completely at low temperature, indicating a potential barrier to triplet fusion that depends on the local environment and cannot be overcome at 1.4 K. Now, the question arises what causes the correlated variations of the rates k_SF_ and k_del_ in the course of time, as shown in Figure 3e. We assume that the coupling strength is related to the current dihedral angle between the two TDI units. Prolonged irradiation of a single molecule and concomitant heat dissipation in its local environment can lead to a conformational change by which the dihedral angle between the two TDI units changes temporarily. In this scenario, the small orbital overlap between the two π-systems is altered leading to a variation of the composition of the mixed spin state. Because the probability of delayed fluorescence scales with the singlet content of the wave function, an increased singlet contribution enhances triplet fusion (k_del_), whereas greater quintet character promotes triplet dissociation (k_SF_).^18^ Throughout the whole sequence depicted in Figure 3e, the uniform fluorescence lifetime and spectra did prove that electronic coupling in the dimer remained essentially constant. This behavior is consistent with our model, because a change of the dihedral angle will not impact the coupling strength in ^1^(S_1_S_0_) considering that the transition dipole moment of TDI is oriented along the long molecular axis.

## 5 Conclusion

In conclusion, SF between two single molecules has been reported for the first time. The single molecule approach has led to a number of novel insights which would be difficult to obtain at the bulk level. The finding of static and dynamic heterogeneities sheds light on the origin of the omnipresent complexity of the SF process. Fluorescence intensity fluctuations of single dimers allow for accessing spin evolution by purely optical means. Experiments at 1.4 K – rarely reported in the SF field – support the initial formation of a superposition state as well as the multistep nature of the SF process. Overall, this study opens new avenues for further exploration of SF in other organic chromophores at the single-molecule level and promises to provide valuable insights into the photophysics of thermally activated delayed fluorescence molecules, which are known for their very large ISC rates.^51^

## Supplementary Material



## Notes

Synthesis of TDI dimer (Figure S1), NMR characterization (Figure S2–S4), calculation of the electron coupling strength and coherence number, single molecule distributions of spectral and photophysical parameters, single molecule spectra (Figure S5–S10) (PDF)

## References

[1] Smith M. B., Michl J. (2010). Singlet Fission. Chem. Rev..

[2] Smith M. B., Michl J. (2013). Recent Advances in
Singlet Fission. Annu. Rev.
Phys. Chem..

[3] Shockley W., Queisser H. J. (1961). Detailed Balance Limit of Efficiency of p-n Junction
Solar Cells. J. Appl. Phys..

[4] Hanna M. C., Nozik A. J. (2006). Solar Conversion
Efficiency of Photovoltaic and Photoelectrolysis
cells with Carrier Multiplication Absorbers. J. Appl. Phys..

[5] Smyser K. E., Eaves J. D. (2020). Singlet fission
for quantum information and quantum
computing: the parallel JDE model. Sci. Rep.

[6] Scholes G. D. (2023). A molecular
perspective on quantum information. Proc. R.
Soc. A.

[7] Casillas R., Papadopoulos I., Ullrich T., Thiel D., Kunzmann A., Guldi D. M. (2020). Molecular insights and concepts to engineer singlet
fission energy conversion devices. Energy Environ.
Sci..

[8] Miyata K., Conrad-Burton F. S., Geyer F. L., Zhu X.-Y. (2019). Triplet Pair States
in Singlet Fission. Chem. Rev..

[9] Casanova D. (2018). Theoretical
Modeling of Singlet Fission. Chem. Rev..

[10] Chan W.-L., Berkelbach T. C., Provorse M. R., Monahan N. R., Tritsch J. R., Hybertsen M. S., Reichman D. R., Gao J., Zhu X.-Y. (2013). The quantum
coherent mechanism for singlet fission: experiment and theory. Acc. Chem. Res..

[11] Young R. M., Wasielewski M. R. (2020). Mixed Electronic
States in Molecular Dimers: Connecting
Singlet Fission, Excimer Formation, and Symmetry-Breaking Charge Transfer. Acc. Chem. Res..

[12] Fuemmeler E. G., Sanders S. N., Pun A. B., Kumarasamy E., Zeng T., Miyata K., Steigerwald M. L., Zhu X.-Y., Sfeir M. Y., Campos L. M., Ananth N. (2016). A Direct Mechanism
of Ultrafast Intramolecular Singlet Fission in Pentacene Dimers. ACS Cent. Sci..

[13] Burdett J. J., Bardeen C. J. (2012). Quantum beats in
crystalline tetracene delayed fluorescence
due to triplet pair coherences produced by direct singlet fission. J. Am. Chem. Soc..

[14] Chan W.-L., Ligges M., Jailaubekov A., Kaake L., Miaja-Avila L., Zhu X.-Y. (2011). Observing the multiexciton
state in singlet fission
and ensuing ultrafast multielectron transfer. Science.

[15] Stern H. L., Cheminal A., Yost S. R., Broch K., Bayliss S. L., Chen K., Tabachnyk M., Thorley K., Greenham N., Hodgkiss J. M., Anthony J., Head-Gordon M., Musser A. J., Rao A., Friend R. H. (2017). Vibronically coherent
ultrafast triplet-pair formation and subsequent thermally activated
dissociation control efficient endothermic singlet fission. Nat. Chem..

[16] Margulies E. A., Logsdon J. L., Miller C. E., Ma L., Simonoff E., Young R. M., Schatz G. C., Wasielewski M. R. (2017). Direct
Observation of a Charge-Transfer State Preceding High-Yield Singlet
Fission in Terrylenediimide Thin Films. J. Am.
Chem. Soc..

[17] Ullrich T., Munz D., Guldi D. M. (2021). Unconventional singlet fission materials. Chem. Soc. Rev..

[18] Musser A. J., Clark J. (2019). Triplet-Pair States
in Organic Semiconductors. Annu. Rev. Phys.
Chem..

[19] Greißel P. M., Schroeder Z. W., Thiel D., Ferguson M. J., Clark T., Guldi D. M., Tykwinski R. R. (2024). Controlling Interchromophore Coupling
in Diamantane-Linked Pentacene Dimers To Create a “Binary”
Pair. J. Am. Chem. Soc..

[20] Millington O., Montanaro S., Sharma A., Dowland S. A., Winkel J., Grüne J., Leventis A., Bennett T., Shaikh J., Greenham N., Rao A., Bronstein H. (2024). The Interplay
of Strongly and Weakly Exchange-Coupled Triplet Pairs in Intramolecular
Singlet Fission. J. Am. Chem. Soc..

[21] Kim J., Teo H. T., Hong Y., Cha H., Kim W., Chi C., Kim D. (2024). Elucidating Singlet-Fission-Born Multiexciton Dynamics
via Molecular Engineering: A Dilution Principle Extended to Quintet
Triplet Pair. J. Am. Chem. Soc..

[22] Greißel P. M., Thiel D., Gotfredsen H., Chen L., Krug M., Papadopoulos I., Miskolzie M., Torres T., Clark T., Bro̷ndsted Nielsen M., Tykwinski R. R., Guldi D. M. (2024). Intramolecular Triplet Diffusion Facilitates Triplet
Dissociation in a Pentacene Hexamer. Angew.
Chem., Int. Ed..

[23] Basel B. S., Zirzlmeier J., Hetzer C., Phelan B. T., Krzyaniak M. D., Reddy S. R., Coto P. B., Horwitz N. E., Young R. M., White F. J., Hampel F., Clark T., Thoss M., Tykwinski R. R., Wasielewski M. R., Guldi D. M. (2017). Unified model for
singlet fission within a non-conjugated covalent pentacene dimer. Nat. Commun..

[24] Tayebjee M. J. Y., Sanders S. N., Kumarasamy E., Campos L. M., Sfeir M. Y., McCamey D. R. (2017). Quintet multiexciton
dynamics in singlet fission. Nat. Phys..

[25] Thiel D., Gotfredsen H., Greißel P. M., Chen L., Krug M., Papadopoulos I., Ferguson M. J., Torres T., Clark T., Neiss C., Görling A., Nielsen M. B., Tykwinski R. R., Guldi D. M. (2025). Interplay between Through-Space and Through-Bond Electronic
Coupling in Singlet Fission. J. Am. Chem. Soc..

[26] Pensack R. D., Ostroumov E. E., Tilley A. J., Mazza S., Grieco C., Thorley K. J., Asbury J. B., Seferos D. S., Anthony J. E., Scholes G. D. (2016). Observation of Two Triplet-Pair Intermediates in Singlet
Exciton Fission. J. Phys. Chem. Lett..

[27] Collins M. I., McCamey D. R., Tayebjee M. J. Y. (2019). Fluctuating exchange interactions
enable quintet multiexciton formation in singlet fission. J. Chem. Phys..

[28] Chen M., Coleman A. F., Young R. M., Wasielewski M. R. (2021). Interplay
between Intermolecular and Intramolecular Singlet Fission in Thin
Films of a Covalently Linked Terrylenediimide Dimer. J. Phys. Chem. C.

[29] Chen M., Shin J. Y., Young R. M., Wasielewski M. R. (2020). Tuning
the charge transfer character of the multiexciton state in singlet
fission. J. Chem. Phys..

[30] Chen M., Krzyaniak M. D., Nelson J. N., Bae Y. J., Harvey S. M., Schaller R. D., Young R. M., Wasielewski M. R. (2019). Quintet-triplet
mixing determines the fate of the multiexciton state produced by singlet
fission in a terrylenediimide dimer at room temperature. Proc. Natl. Acad. Sci..

[31] Schultz J. D., Shin J. Y., Chen M., O’Connor J. P., Young R. M., Ratner M. A., Wasielewski M. R. (2021). Influence
of Vibronic Coupling on Ultrafast Singlet Fission in a Linear Terrylenediimide
Dimer. J. Am. Chem. Soc..

[32] Chen M., Bae Y. J., Mauck C. M., Mandal A., Young R. M., Wasielewski M. R. (2018). Singlet
Fission in Covalent Terrylenediimide Dimers:
Probing the Nature of the Multiexciton State Using Femtosecond Mid-Infrared
Spectroscopy. J. Am. Chem. Soc..

[33] Mandal A., Chen M., Foszcz E. D., Schultz J. D., Kearns N. M., Young R. M., Zanni M. T., Wasielewski M. R. (2018). Two-Dimensional
Electronic Spectroscopy Reveals Excitation Energy-Dependent State
Mixing during Singlet Fission in a Terrylenediimide Dimer. J. Am. Chem. Soc..

[34] Margulies E. A., Miller C. E., Wu Y., Ma L., Schatz G. C., Young R. M., Wasielewski M. R. (2016). Enabling
singlet fission by controlling
intramolecular charge transfer in π-stacked covalent terrylenediimide
dimers. Nat. Chem..

[35] Bernard J., Fleury L., Talon H., Orrit M. (1993). Photon bunching in
the fluorescence from single molecules: A probe for intersystem crossing. J. Chem. Phys..

[36] Basché T., Moerner W. E., Orrit M., Talon H. (1992). Photon antibunching
in the fluorescence of a single dye molecule trapped in a solid. Phys. Rev. Lett..

[37] Fleury L., Segura J., Zumofen G., Hecht B., Wild U. P. (2000). Nonclassical
photon statistics in single-molecule fluorescence at room temperature. Phys. Rev. Lett..

[38] Chen Q., Thoms S., Stöttinger S., Schollmeyer D., Müllen K., Narita A., Basché T. (2019). Dibenzo­[hi,st]­ovalene
as Highly Luminescent Nanographene: Efficient Synthesis via Photochemical
Cyclodehydroiodination, Optoelectronic Properties, and Single-Molecule
Spectroscopy. J. Am. Chem. Soc..

[39] Verhart N. R., Navarro P., Faez S., Orrit M. (2016). Intersystem crossing
rates of single perylene molecules in ortho-dichlorobenzene. Phys. Chem. Chem. Phys..

[40] Mais S., Tittel J., Basché T., Bräuchle C., Göhde W., Fuchs H., Müller G., Müllen K. (1997). Terrylenediimide: A Novel Fluorophore for Single-Molecule
Spectroscopy and Microscopy from 1.4 K to Room Temperature. J. Phys. Chem. A.

[41] Métivier R., Nolde F., Müllen K., Basché T. (2007). Electronic
excitation energy transfer between two single molecules embedded in
a polymer host. Phys. Rev. Lett..

[42] Spano F. C., Yamagata H. (2011). Vibronic coupling in
J-aggregates and beyond: a direct
means of determining the exciton coherence length from the photoluminescence
spectrum. J. Phys. Chem. B.

[43] Diehl F. P., Roos C., Duymaz A., Lunkenheimer B., Köhn A., Basché T. (2014). Emergence
of Coherence through Variation
of Intermolecular Distances in a Series of Molecular Dimers. J. Phys. Chem. Lett..

[44] Nolde F., Pisula W., Müller S., Kohl C., Müllen K. (2006). Synthesis
and Self-Organization of Core-Extended Perylene Tetracarboxdiimides
with Branched Alkyl Substituents. Chem. Mater..

[45] Novotny, L. ; Hecht, B. Principles of Nano-Optics; Cambridge University Press, 2012.

[46] Loudon, R. The quantum theory of light, 3rd ed.; Oxford Science Publications; Oxford University Press, 2000.

[47] Lippitz M., Hübner C. G., Christ T., Eichner H., Bordat P., Herrmann A., Müllen K., Basché T. (2004). Coherent electronic
coupling versus localization in individual molecular dimers. Phys. Rev. Lett..

[48] Haase M., Hübner C. G., Nolde F., Müllen K., Basché T. (2011). Photoblinking
and photobleaching of rylene diimide
dyes. Phys. Chem. Chem. Phys..

[49] Maslennikov D. R., Maimaris M., Ning H., Zheng X., Mondal N., Bruevich V. V., Pratik S. M., Dong Y., Tisch J. W. G., Musser A. J., Podzorov V., Bredas J.-L., Coropceanu V., Bakulin A. A. (2025). Interplay between Mixed and Pure Exciton States Controls
Singlet Fission in Rubrene Single Crystals. J. Am. Chem. Soc..

[50] Majumder K., Mukherjee S., Panjwani N. A., Lee J., Bittl R., Kim W., Patil S., Musser A. J. (2023). Controlling Intramolecular Singlet
Fission Dynamics via Torsional Modulation of Through-Bond versus Through-Space
Couplings. J. Am. Chem. Soc..

[51] Samanta P. K., Kim D., Coropceanu V., Brédas J.-L. (2017). Up-Conversion Intersystem Crossing
Rates in Organic Emitters for Thermally Activated Delayed Fluorescence:
Impact of the Nature of Singlet vs Triplet Excited States. J. Am. Chem. Soc..

