# Supplementary material for: Singlet Fission among Two Single Molecules
Source: J Am Chem Soc. 2026 Jun 15;148(25):26343–52. doi: 10.1021/jacs.6c05963 (PMC13339131; doi:10.1021/jacs.6c05963)
Supplement: Supplementary file 1 [file ja6c05963_si_001.pdf]

Supporting Information

Singlet Fission among Two Single Molecules

Sumanta Paul[1], Oleksandr Yampolskyy[1], Zehua Wu[1], Klaus Müllen[1,2], Thomas Basché[1,*]

[1]Department of Chemistry, Johannes Gutenberg-Universität, 55099 Mainz, Germany

[2]Max Planck-Institute for Polymer Research, 55099 Mainz, Germany

*Corresponding author. Email: thomas.basche@uni-mainz.de

S1. Synthesis of the TDI dimer

The synthesis of the TDI dimer was achieved through a two-step process, following the same procedure previously reported in the literature.[1] In the first step, Suzuki coupling between compounds 1 and 2 yielded compound 3 with a 70% yield. This was followed by a base-induced dehydrogenation reaction using K_2CO_3, resulting in the formation of the fully conjugated TDI dimer skeleton. The crude product was purified by column chromatography on SiO_2 (CH_2Cl_2 / methanol=50:1) followed by size exclusion chromatography (Bio-Rad Bio-Beads, S-X1, THF). ^1H NMR (500 MHz, $C_2D_2Cl_4$, 403K) δ 8.65 (8H, br, ArH), 8.51 (16H, br, ArH), 5.20 (2H, br, ArH), 2.29 (8H, m, CH_2), 1.97 (8H, m, CH_2), 1.33 (30H, m, CH_2), 0.90 (12H, t, J= 6.5 Hz, CH_3). HRMS MALDI-TOF (TCNQ): calculated for $C_{98}H_{84}N_4O_8$ m/z = 1446.6446 found m/z = 1446.6385. MP > 400 °C.

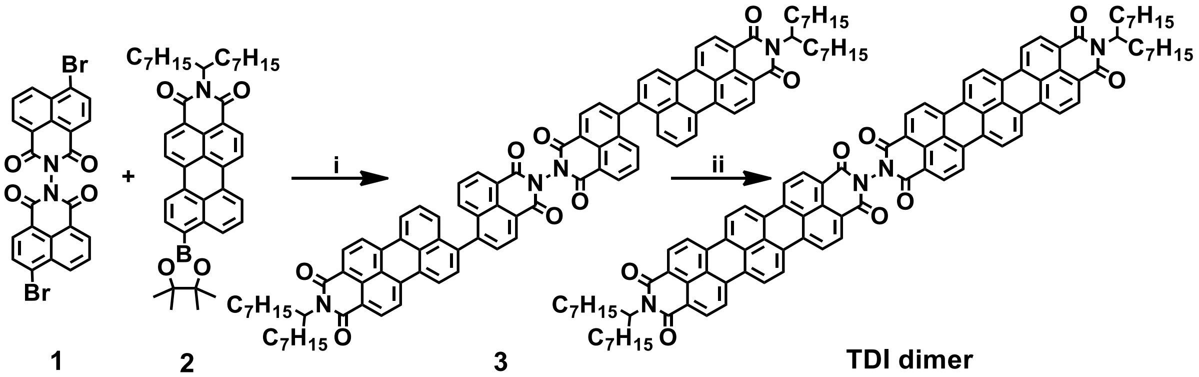

1 2 3 TDI dimer

Figure S1. Synthesis of the TDI dimer. Reaction conditions: (i) Tetrakis (triphenylphosphine) palladium (0), K_2CO_3, toluene, 100°C, 12 h, 70%. (ii) K_2CO_3, ethanolamine, 160°C, 12 h, 10%.

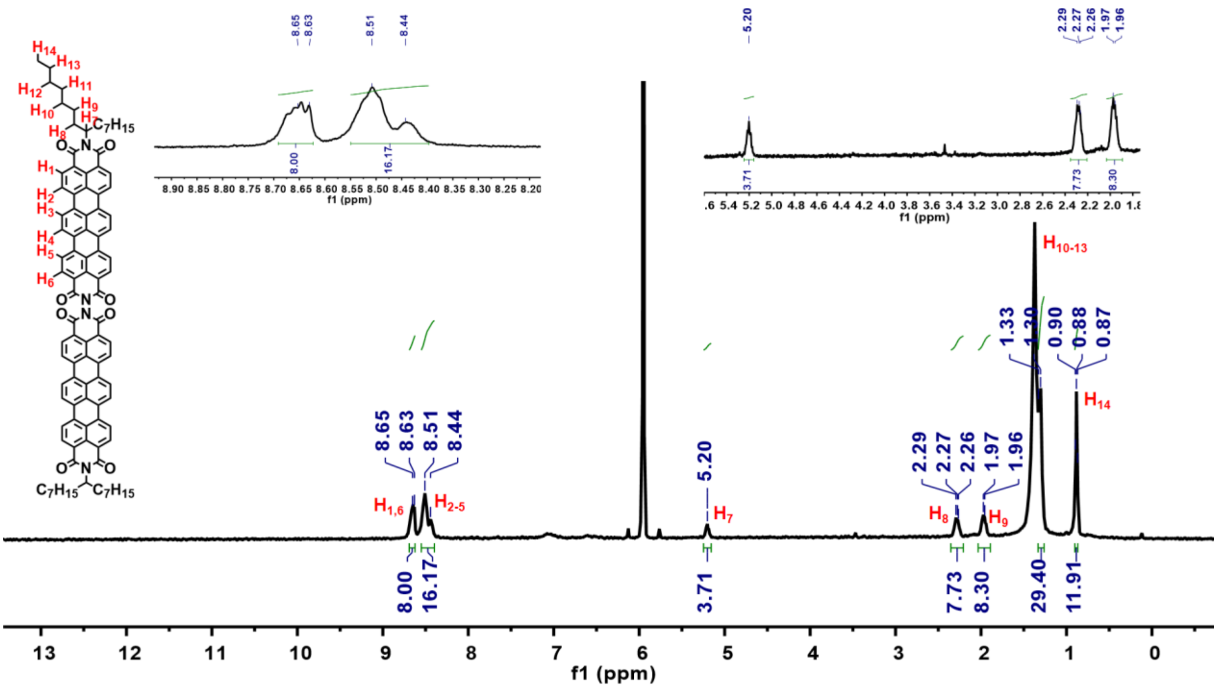

Figure S2. ^{1}H NMR spectrum of TDI dimer (500 MHz, $C_2D_2Cl_4$, 403K).

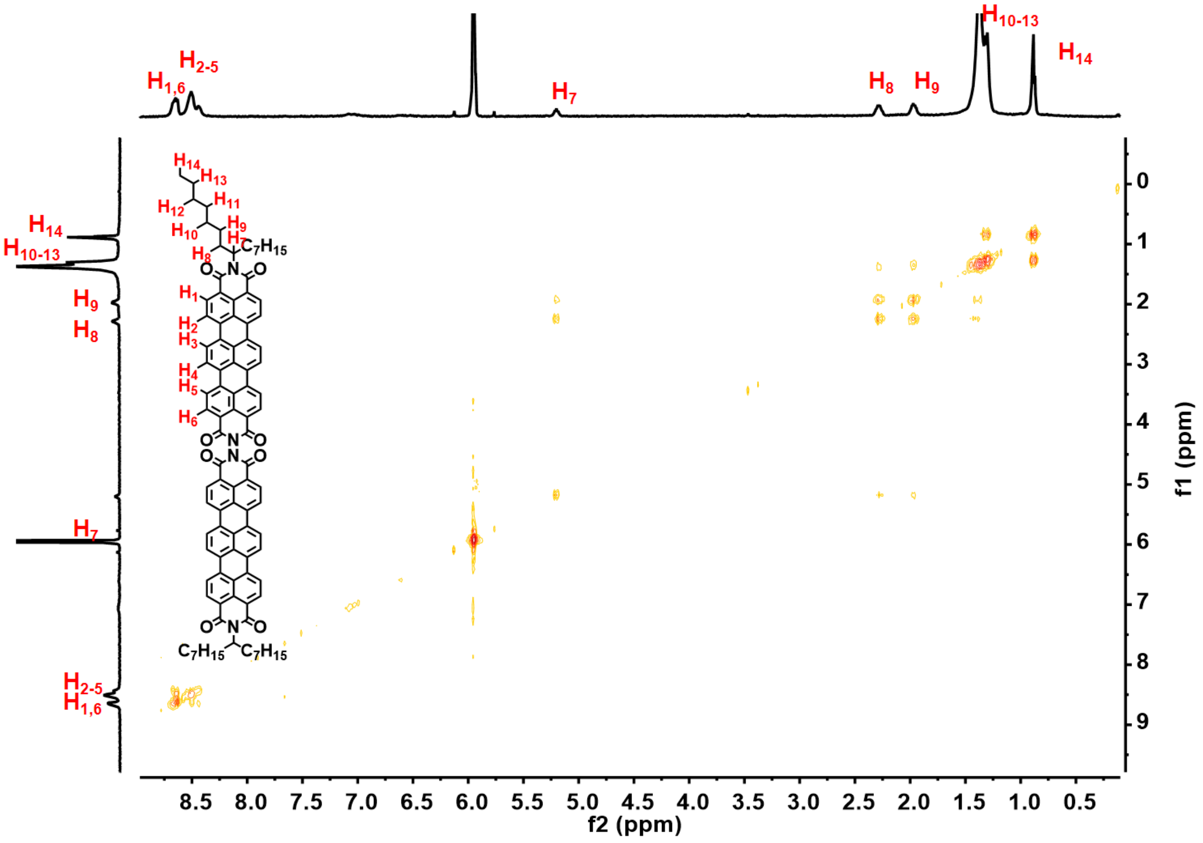

Figure S3. ^{1}H-^{1}H COSY spectrum of TDI dimer (500 MHz, $C_2D_2Cl_4$, 403K).

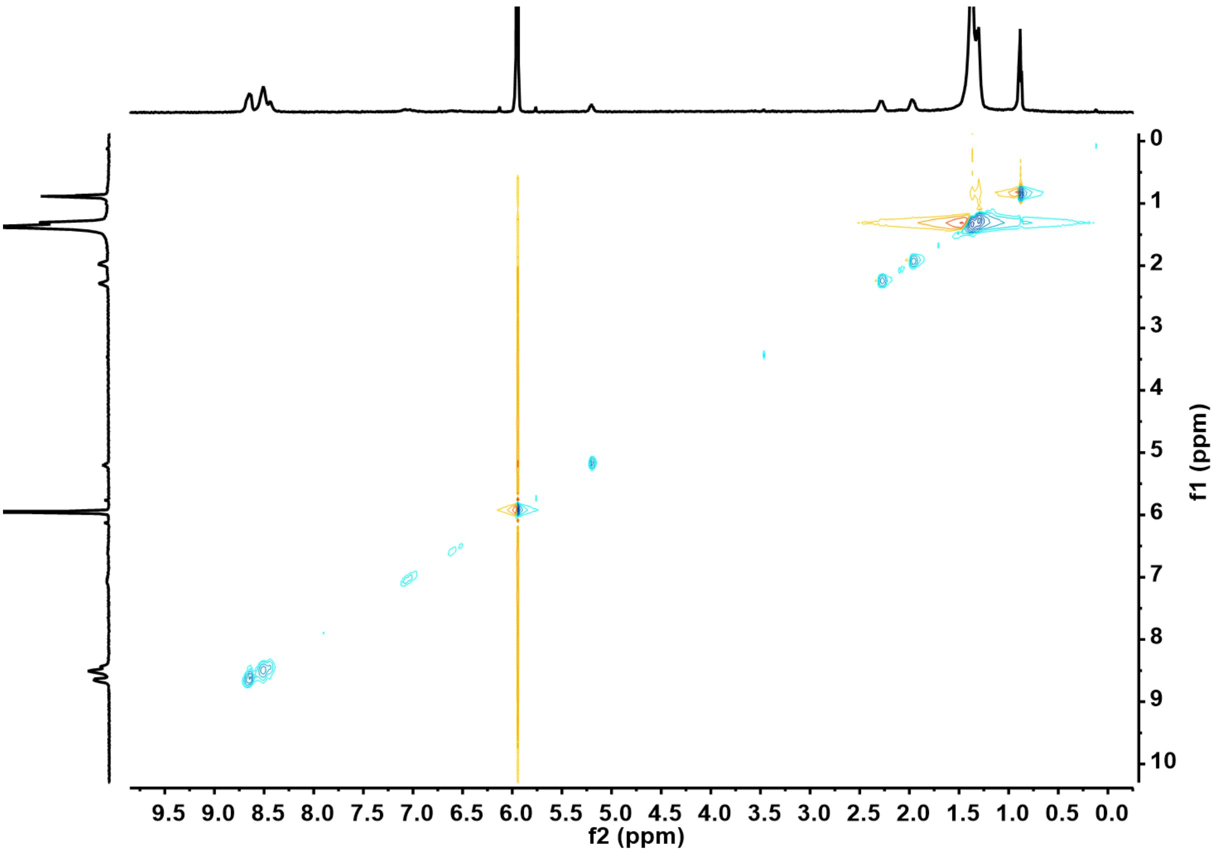

Figure S4. ^{1}H-^{1}H NOESY spectrum of the TDI dimer (500 MHz, $C_2D_2Cl_4$, 403K).

S2. Calculation of the electron coupling strength and coherence number

Assuming through space electrostatic dipole-dipole coupling, the coupling strength V in the TDI dimer was estimated by equation 1:

$$V = \frac{1}{4\,\pi\,\varepsilon_0}\frac{|\mu^2|\,\kappa}{R^3} \qquad (1)$$

where μ = 11 Debye is the transition dipole moment of the TDI monomer in toluene, κ = 2 the orientational factor and R = 1.73 nm the centre-to-centre distance in the dimer. V was found to be 240 cm^{-1}.

As has been shown by Spano et al.[2], for J-type aggregates with a dominating vibrational mode and in the presence of disorder, the coherence number N_{coh} at T > 0 K can be closely approximated by equation 2:

$$\frac{I_{0,0}}{I_{0,1}} = \frac{N_{coh}}{S} \qquad (2)$$

$I_{0,0}$ and $I_{0,1}$ are the integrated intensities of the 0,0-transition and 0,1-transition in the fluorescence spectrum and S is the Huang-Rhys factor of the coupled vibrational mode. From the fluorescence spectrum of the TDI dimer in toluene we obtain $I_{0,0}/I_{0,1}$ = 1.9 and from the TDI monomer fluorescence spectrum S = 0.74, a typical value for a rigid organic dye molecule. Putting these numbers into equation 2, we get N_{coh} = 1.4. Qualitatively, this number is related to the degree of delocalization of the electronic excitation in the TDI dimer in the presence of disorder and at finite temperature. In terms of radiative rates, N_{coh} is given by the ratio of the dimer to monomer radiative rates.[3] Using this relation, we find a radiative rate in the TDI dimer of $k_{rad}^{Dimer} = k_{rad}^{Monomer} \cdot 1.4 = 2.9 \cdot 10^8\ s^{-1}$ which is in full agreement with the radiative rate obtained from the quantum yield and lifetime.

S3. Single molecule spectra and distributions of spectral and photophysical parameters

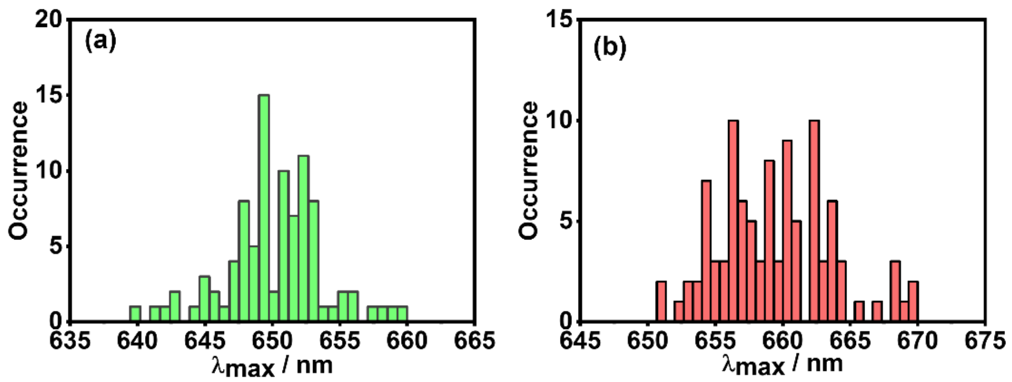

Figure S5. Distributions of fluorescence peak positions of individual TDI monomers (a) and dimers (b). The average values are given in table 2.

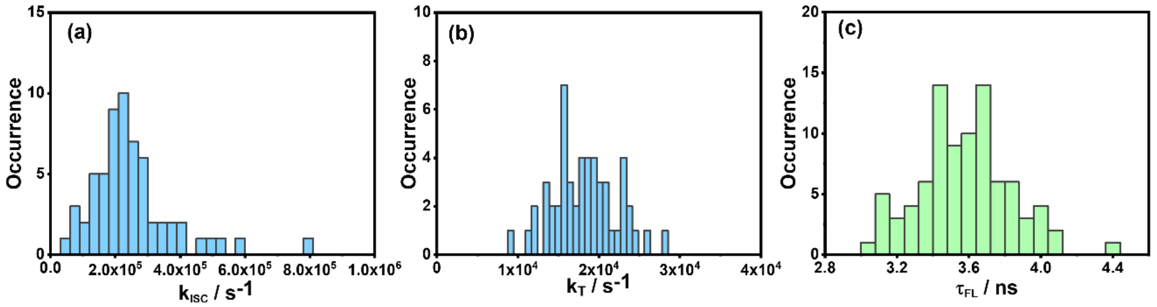

Figure S6. Distributions of the ISC rate (a), triplet decay rate (b) and fluorescence lifetime (c) of the TDI monomer. The average values are given in table 2.

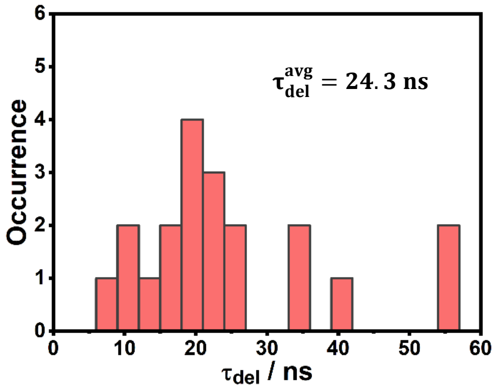

Figure S7. Distribution of lifetimes of delayed fluorescence for the TDI dimer after pulsed excitation.

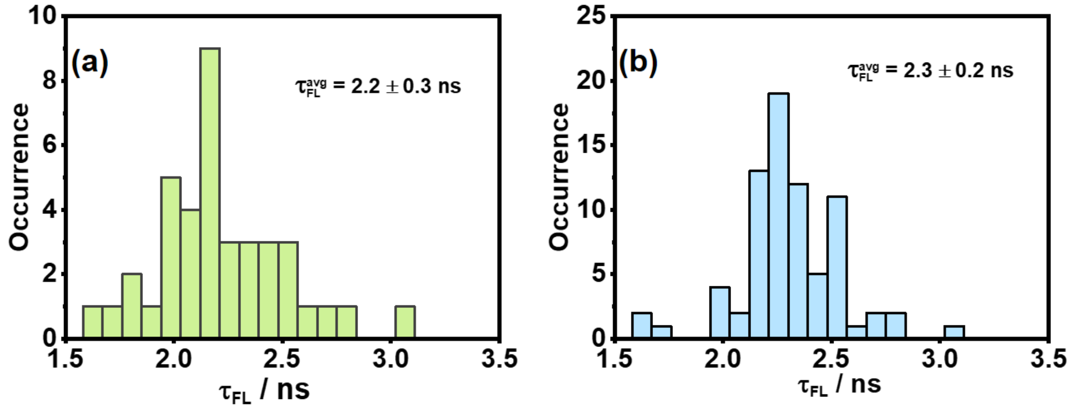

Figure S8. Distributions of the fluorescence lifetime (τ_{FL}) of the TDI dimer derived from autocorrelation analysis after continuous wave excitation (a) or from fluorescence decay measurements after pulsed excitation (b).

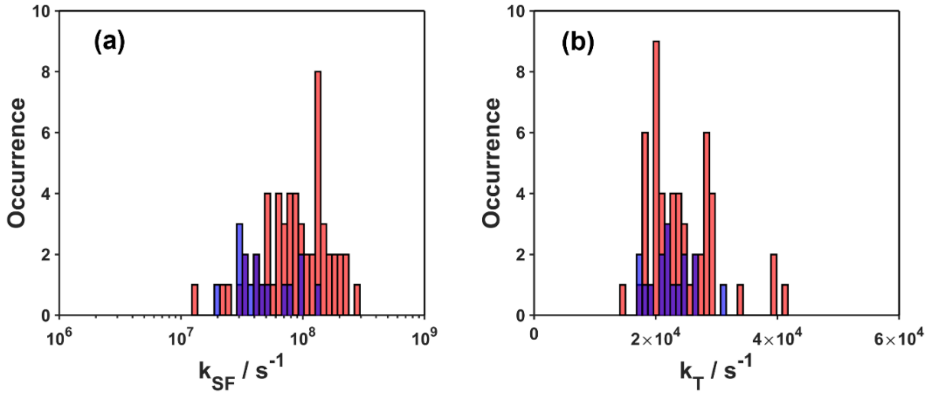

Figure S9. Distributions of the rates k_{SF} (a) and k_T (b) for TDI dimers which showed only prompt fluorescence (red) or prompt as well as delayed fluorescence (blue) after pulsed excitation. The average values are: Prompt fluorescence: $k_{SF} = 1.0 \pm 0.6 \cdot 10^8\ s^{-1}$, $k_T = 2.4 \pm 0.6\ \cdot 10^4\ s^{-1}$; prompt and delayed fluorescence: $k_{SF} = 6.0 \pm 3 \cdot 10^7\ s^{-1}$, $k_T = 2.2 \pm 0.4 \cdot 10^4\ s^{-1}$.

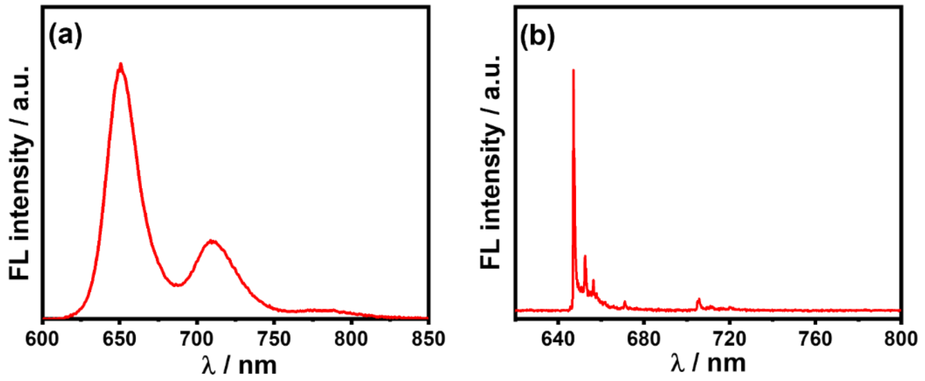

Figure S10. Fluorescence spectra of single TDI monomers at room temperature (a) and at T = 1.4 K (b). The comparison shows how the spectrum develops from room temperature to 1.4 K at which sharp zero-phonon lines (ZPL) are observed.

REFERENCES

1. Chen, M.; Krzyaniak, M. D.; Nelson, J. N.; Bae, Y. J.; Harvey, S. M.; Schaller, R. D.; Young, R. M.; Wasielewski, M. R. Quintet-triplet mixing determines the fate of the multiexciton state produced by singlet fission in a terrylenediimide dimer at room temperature. *Proc. Natl. Acad. Sci.* **2019**, *116*, 8178−8183.
2. Spano, F. C.; Yamagata, H. Vibronic coupling in J-aggregates and beyond: a direct means of determining the exciton coherence length from the photoluminescence spectrum. *J. Phys. Chem. B,* **2011,** *115,* 5133–5143.
3. Meier, T.; Zhao, Y.; Chernyak, V.; Mukamel, S. Polarons, localization, and excitonic coherence in superradiance of biological antenna complexes. *J. Chem. Phys*. **1997**, *107*, 3876-3893.
